# Suicide and Changes in Expression of Neuronal miRNA Predicted by an Algorithm Search through miRNA Databases

**DOI:** 10.3390/genes13040562

**Published:** 2022-03-23

**Authors:** Alja Videtič Paska, Urban Alič, Tomaž Zupanc, Katarina Kouter

**Affiliations:** 1 Institute of Biochemistry and Molecular Genetics, Faculty of Medicine, University of Ljubljana, 1000 Ljubljana, Slovenia; alja.videtic@mf.uni-lj.si; 2 Faculty of Medicine, University of Ljubljana, 1000 Ljubljana, Slovenia; urbanalich@gmail.com; 3 Institute of Forensic Medicine, Faculty of Medicine, University of Ljubljana, 1000 Ljubljana, Slovenia; tomaz.zupanc@mf.uni-lj.si

**Keywords:** psychiatry, epigenetics, Brodmann area 10, non-coding RNA, suicidal behaviour, micro RNA

## Abstract

Suicide is multifactorial and polygenic phenotype, affected by environmental and genetic factors. Among epigenetic mechanisms, miRNAs have been studied, but so far no very concise results exist. To overcome limitations of candidate miRNA and whole genome sequencing approaches, we created an in silico analysis algorithm that would help select the best suitable miRNAs that target the most interesting genes associated with suicidality. We used databases/web algorithms DIANA microT, miRDB, miRmap, miRWalk, and TargetScan and candidate genes *SLC6A4*, *HTR1A*, *BDNF*, *NR3C1*, *ZNF714*, and *NRIP3*. Based on a prediction algorithm, we have chosen miRNAs that are targeting regulation of the genes listed, and are at the same time being expressed in the brain. The highest ranking scores were obtained for hsa-miR-4516, hsa-miR-3135b, hsa-miR-124-3p, hsa-miR-129-5p, hsa-miR-27b-3p, hsa-miR-381-3p, hsa-miR-4286. Expression of these miRNAs was tested in the brain tissue of 40 suicide completers and controls, and hsa-miR-4516 and hsa-miR-381-3p showed a trend for statistical significance. We also checked the expression of the target genes of these miRNAs, and for *NR3C1* expression was lower in suicide completers compared to controls, which is in accordance with the available literature results. To determine the miRNAs that are most suitable for further suicidality research, more studies, combining in silico analysis and wet lab experiments, should be performed.

## 1. Introduction

Suicide is a major public health issue, with more than 700,000 or 1.3% of deaths every year [1]. Although public awareness regarding suicidality has been rising over the past years, the goal of the international public health community to reduce the global suicide mortality rate by one third by 2030 will not be achieved [2]. In order to address the issue of suicidality comprehensively, many actions have been taken, and the available literature addressing the suicidality aetiology is growing every year. Suicidality is a complex behavioural phenotype, affected by many different risk and protective factors, including social, cultural, psychological, clinical, biological and environmental factors. To better understand the suicide risk, several different models have been proposed. One of them is the biopsychosocial model, which takes into account all of the previously listed factors and how they are connected [3]. Through family, twin and adoption studies, it has been identified that both environmental and genetic components make a significant contribution to the suicidality phenotype [4,5,6,7]. Over the past four decades, the genetic contribution to suicidality has been researched mostly on a DNA level, addressing the differences in genotype and allele frequencies distributions between different shapes of suicidality. The results of these studies were further evaluated in meta-analyses of several candidate genes for suicidality. These genes were recognised inside the neurotransmitter signaling pathways that were identified through studies of the neurobiological background of suicidality. The most dissected has been the serotonergic system and the polymorphisms in the genes for serotonin transporter (SLC6A4) [8], serotonin receptors (HTR), and tryptophan hydroxylase [9,10]. The physiological functions of serotonin are involved in the regulation of the development and plasticity of neural circuits, and are closely associated with actions of brain-derived neurotrophic factor (BDNF), a neurotrophin and member of a nervous growth factor family. BDNF, another important candidate gene for suicidality, promotes survival and differentiation of serotonergic neurons, while on the other hand elevated synaptic levels of serotonin positively regulate transcription of *BDNF* [11,12]. The functional polymorphism of *BDNF* has been researched extensively and it was associated with the possibility for increased risk for suicidal behaviour [13]. The results of the studies of candidate genes and polymorphisms on suicidality did not provide a clear answer to the causality of it, but it became evident that suicidality has a multifactorial and polygenic background. The search for novel genes associated with suicidality was advanced through genome-wide association studies interrogating hundreds of thousands of polymorphisms. However, this attempt did not provide consistent results, and previously identified candidate genes were frequently failing to pass rigorous reproducibility and significance testing [3,14].

Due to the multifaceted nature of suicidality and the insufficient clarification of the genetic background of suicidality, the studies were further oriented towards the simultaneous investigation of several factors and, in this case, the epigenetic studies aimed to link the environmental and genetic factors. Among the genes in epigenetic studies, very notable results were obtained for glucocorticoid receptor gene (*NR3C1*). It is a part of the negative loop regulating the stress response axis hypothalamus-pituitary-adrenal gland (HPA), often disturbed in mental disorders and suicidality [15]. In the brains of the suicide completers, lower expression of the *NR3C1* was determined, and this could be the cause for the higher activity of the HPA axis [15,16]. Epigenetic regulation of the *NR3C1* was also determined in the subjects that experienced early adverse events [17], pointing out the possible environmental effects on gene expression.

Through epigenetics, we study the differences in gene expression patterns, which are under very precise spatiotemporal control. The three most common epigenetic mechanisms are noncoding RNA, DNA methylation and modifications of histone proteins. Micro RNAs (miRNAs) belong to the short noncoding RNAs and are about 22 nucleotides in length. In humans, there are more than 2500 different miRNAs whose presence was also experimentally confirmed [18,19]. However, some estimates predict, in human genomes, the existence of up to one million corresponding sequences that could, after transcription, enter into the process of miRNA biogenesis [20]. MiRNA biogenesis is a multistep process that starts in the nucleus and completes in the cytoplasm with mature miRNAs [21]. Biogenesis initiates with RNA polymerase II that synthesises the first primary pri-miRNA transcript of about 70 nucleotides. The pri-miRNA has the typical hairpin loop structure, which is the substrate for microprocessor. The latter is a heterotrimeric complex protein that contains one molecule of the Drosha endonuclease and two molecules of its partner protein, DGCR8, and it catalyses the first step of enzymatic cleavage of pri-miRNA to pre-miRNA [16,22,23]. After cleavage, the pre-miRNA is transported to the cytoplasm via a transporter protein exportin 5. In the cytoplasm, further enzymatic cleavage of the hairpin structured pre-miRNA is catalysed by endoribonuclease Dicer. This results in a double-stranded RNA molecule, with mature miRNA and its complementary, passenger strand [23]. In most cases, only one strand of the double-stranded molecule is functionally active and becomes miRNA, while the other is degraded; however, sometimes both strands can be retained and become miRNAs [23,24]. Mature miRNA, bound to its complementary strand, with some assistance from chaperone proteins, is eventually loaded into an Argonaute (Ago) protein. At this point, the selection of the miRNA that will serve as the guide strand of the of the RNA-induced silencing complex (RISC) is performed, while the complementary strand is degraded [23]. The role of the RISC molecule is a recognition of the short sequence within the 3′ untranslated region (3′UTR) of target genes, to which miRNA molecules bind and cause messenger RNA (mRNA) silencing [16] through three possible different mechanisms. These are either degradation of mRNA through endoribonuclease activity of protein Ago3, which is part of the RISC [16,21,25], shortening of the polyadenilated tail at the 3′-end of the mRNA leading to lower stability of mRNA and accelerated degradation with cytoplasmic RNases [21], or inhibition of the ribosome binding to the mRNA, resulting in a reduction in translation efficiency [21,25].

The miRNAs’ main function is, therefore, post-transcriptional gene regulation at the level of mRNA, and miRNAs thus act as an important epigenetic mechanism of gene downregulation [26]. MiRNA molecules are of specific interest in the study of psychiatric disorders, since neuronal miRNA represent 70% of the total miRNAs in our body, and they are involved in neurogenesis and neuroplasticity [27]; hence, they represent a promising biomarker potential. However, one target mRNA can be regulated through several miRNAs, while one particular miRNA can regulate several different mRNAs [16,19,22,28]. This makes the identification of the potential miRNAs as a biomarker a quite elaborate task, but the use of modern in silico approaches could facilitate the work greatly.

In order for miRNAs to accomplish their goal, a specific intermolecular interaction occurs between miRNA and mRNA molecules. The probability and strength of such an interaction depends primarily on the nucleotide sequence of both molecules. With in silico analysis, it is possible to evaluate miRNAs and their role in gene expression. Several different databases/web algorithms, such as DIANA microT [29], miRDB [30], miRmap [31], miRWalk [32], and TargetScan [33], which collect and store data on miRNA are available. However, these databases use different algorithms and therefore the annotation of miRNA-mRNA pairs can vary. In order to minimise the type I and type II errors, it is advisable to use more independent sources, since a significant number of miRNA–mRNA interactions are false positives or negatives [34].

Since different databases do not necessarily predict the action of specific miRNAs in the same way, and because miRNAs are very abundant in the brain where they fine-tune explicit gene expression, we aimed to design an algorithm that would enable the evaluation the robustness of the results of particular miRNAs targeting and their expression in the brain from several distinct data sources (databases) [29,30,31,32,33,35] which could serve as potential biomarkers in suicidality. As target genes, we have selected several already well recognised candidate genes for suicidality (*SLC6A4*, *HTR1A*, *BDNF*, *NR3C1*), identified through the search of rather comprehensive available literature results, and additionally two genes (*ZNF714*, *NRIP3*) that we have identified as differentially methylated and expressed in a study on genome-wide DNA methylation in the brains of suicide completers [36]. We performed the miRNA isolation and quantitative real-time PCR (qPCR) analysis on the samples of Brodmann area 10 (BA10) on suicide completers and controls.

## 2. Materials and Methods

### 2.1. Subjects

Altogether our study included 40 male subjects, 20 suicide completers and 20 control group subjects. The cause of death, determined by the medical examiner after completed autopsy, was hanging for suicide victims and sudden cardiac arrest for the control group subjects. Exclusion criteria (in addition to other causes of death and female sex) were age over 65 (to avoid age-related neurodegeneration) and alcohol dependence syndrome, which could cause age- or alcohol-related epigenetic changes, and insufficient body preservation that could be associated with poor RNA isolation yields and integrity. In blood and urine, basic toxicology and alcoholometry screening were performed. To rule out alcohol misuse status, histological changes in the liver that could be attributed exclusively to excessive alcohol consumption (fatty liver disease with >60% of hepatocytes showing macrovesicular hepatocyte fatty change, alcohol hepatitis and hepatic cirrhosis) with simultaneous exclusion of causal factors for histologic changes in the liver (abnormalities of hepatic tissue due to obesity, diabetes and/or congestive heart failure) were determined. Average age, postmortem interval and RIN value of suicide completers and control group subjects are presented in Table 1. Detailed subject information can be found in the Supplementary Table S1. Imaging and molecular studies have implicated the prefrontal cortex in suicidality. Brodmann area 10 is a region located in the frontopolar/rostrolateral prefrontal cortex, and is thought to be involved in memory recall, cognitive awareness and various executive functions [37]. Brain tissue samples of prefrontal cortex (BA10) were collected during routine autopsy performed at the Institute of Forensic Medicine, Faculty of Medicine, University of Ljubljana. All samples were collected by the same medical examiner, immediately frozen in liquid nitrogen and stored at −80 °C until further processing. The institutional review board of the Slovenian National Medical Ethics Committee approved our study, code 0120-392/2020-6.

### 2.2. Algorithm Design and in Silico Selection of miRNAs

To select the miRNAs for biochemical analysis, we designed a sorting algorithm that enabled us to order a list of miRNAs into a sequence based on the probability of having differential expression in suicide completers. This probability was evaluated by calculating a score for each miRNA which consisted of two components: target interaction score (from in silico target interaction analysis) and expression score (from in silico miRNA expression analysis). Each of those two components on average provided 50% to the total score.

For target interaction analysis, we selected six target genes that are characterised by differential expression in suicide completers (target genes being *BDNF*, *HTR1A*, *SLC6A4*, *NR3C1*, *ZNF714*, and *NRIP3*) based on literature research and results of our previous study [36]. Several online biological databases and algorithms exist, which evaluate which miRNAs are involved in regulation of target gene expression based on molecular interaction with the mRNA. We collected the data from five such biological databases/web algorithms (called databases below): DIANA microT (version 5.0, released in 2013) [29], miRDB (version 6.0, released in 2020) [30], miRmap (version 1.1, released in 2013) [31], miRWalk (version 3.0, released in 2018) [32] and TargetScan (version 7.2, released in 2018) [33]. Each database provided a set of miRNAs that could regulate the expression of a target gene, while some of them also provided a score that evaluated the probability/strength of intermolecular interaction. Table 2 includes the information about the predicted number of miRNAs targeting each of the selected target genes. In total, we collected more than 20,000 data entities, each of them providing an evaluation of a specific interaction between a miRNA and one of the target genes.

As the data from different databases were not given in an identical format, data standardisation was performed. A 4-dimensional vector (containing miRNA identifier, target gene symbol, interaction evaluation, and database name) was selected as the standard data model. The major challenge was to reduce every data entity to a value in the interval (0, 1) where 0 or 1 would imply a low or a high probability of intermolecular interaction, respectively. Firstly, we converted the category given by databases miRWalk and TargetScan into a numerical value: 0 (database has not predicted regulation of target gene); 1 (database has predicted regulation of target gene). Next, the databases miRDB and miRmap provided values on a numerical scale that was two orders of magnitude higher than desired. A linear transformation with a normalisation factor of 0.01 was performed to fit the standard data model. Finally, to correct systemic differences between databases, we added a constant of 0.5 to all values from databases miRDB, miRmap and DIANA microT. This resulted in some values reaching as high as 1.5, but this was still acceptable.

The second component of the score was obtained from in silico expression analysis. Expression of miRNA is not universal throughout the body, each tissue has a specific miRNA expression profile, and this is something that we corrected for to decrease the proportion of false positive matches. A study by Ludwig et al. measured the expression of 1997 miRNAs using RNA-microarray analysis. Authors mapped the miRNA expression profile in 61 different tissues of two human subjects. Furthermore, in the study, the raw experimental data were normalised using variance-stabilised normalisation and corrected for negative values by adding a constant. This sequence of processes rendered the values that were directly used by our algorithm. We selected only the normalised expression data from regions “brain_1” and “brain_cerebral_coretex_2” [35]. These brain regions were selected because they contain BA10, which was the source of the biological samples we studied.

There were a total of 2511 miRNA included in in silico analysis; some of them had full and some only partial data available. For every miRNA, six distinct target interaction scores were calculated, one for each target gene, by adding together the values from all five databases. To determine the interaction score that a miRNA has with the whole group of target genes, we added together interaction scores for every target gene and obtained the overall interaction score represented by a number in the interval (0, 39) (5 databases × 6 genes, 30 values combined). The other part of the total score (expression score) provided values in the interval (0, 10). To ensure that each score component would provide 50% to the total score, we performed a linear transformation on expression data with normalisation factor of 2.4 (0.4 for each gene). By finally adding together the overall target interaction score and normalised expression score, we determine the total score of a miRNA. Based on this score, the sorting algorithm can determine which miRNAs have the highest probability to be involved in gene regulation of (differentially expressed) target genes. For the biochemical analysis, we selected 10 miRNAs with the highest total scores, but later reduced the selection to 7 miRNAs due to qPCR reagent quality issues. Detailed information and algorithm source codes are available at https://github.com/ualich/miRNAtpa (accessed on 21 January 2022).

### 2.3. Isolation and Gene Expression of miRNA and Target Gene mRNA

To isolate RNA, we used 50 mg of frozen and ground BA10 tissue. We isolated both miRNA and mRNA simultaneously using mirVana miRNA Isolation Kit (Thermo Fisher Scientific, Waltham, MA, USA), following the manufacturer instructions. Isolated RNA was quantified using Synergy H4 and 2100 Bioanalyzer (Agilent, Santa Clara, CA, USA). RIN values (mean ± SD) were 7.7 ± 0.22 for suicide completers and 7.26 ± 0.27 for control group subjects.

For miRNA analysis, RNA underwent reverse transcription using TaqMan Advanced miRNA cDNA Synthesis Kit (Thermo Fisher Scientific, Waltham, MA, USA), following the manufacturer instructions. For mRNA analysis, RNA underwent reverse transcription using Maxima H Minus cDNA Synthesis Master Mix kit (Thermo Fisher Scientific, Waltham, MA, USA), following the manufacturer instructions.

Gene expression levels were quantified using qPCR following the MIQE guidelines [38]. All qPCR reactions were run using Viia 7 real-time PCR system and hydrolysis probes (both Thermo Fisher Scientific, Waltham, MA, USA). All hydrolysis probes were validated beforehand and negative control was used for each qPCR. We used has-miR-99a-5p and GAPDH as reference genes for miRNA and mRNA quantificatiohashsa-miR-99a-5p was selected based on the qualities needed for a good reference gene: stable expression level through various tissue, sufficient level of expression in the brain tissue and it ranked low on our algorithm. GAPDH was chosen as it is one of the most commonly used reference genes for RNA gene expression analysis. 

### 2.4. Statistical Analysis

Gene expression data were analysed relative to the corresponding reference gene, following the 2^−ΔΔCq^ method by Livak et al. [39]. Based on the value of sample’s quantification cycle (Cq), we compared relative changes in gene expression between suicide completers and control group subjects. Next, the distribution of data set was assessed using the D’Agostino and Pearson test. As all data did not follow normal distribution, data set values were further analysed using the nonparametric Mann–Whitney U test. The results are presented and considered without correction for multiple testing as the study was explorative in nature. Statistical tests and figures were made using GraphPad Prism version 8.4.3 for Windows (GraphPad Software, San Diego, CA, USA, www.graphpad.com, accessed on 21 January 2022). *p*-values < 0.05 were considered as statistically significant.

## 3. Results

### 3.1. MiRNA Gene Expression

Final miRNA selection was based on the total algorithm score (comprised of interaction scores and expression score). The total score was calculated for all 2511 miRNAs included in in silico analysis. Based on this in silico analysis, we selected seven miRNAs for experimental validation (Table 3).

**Figure 1:**
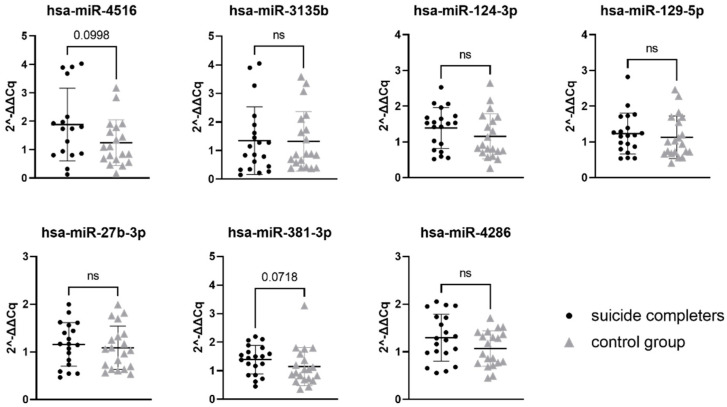
Relative gene expression of selected miRNAs in Brodmann area 10. Data are presented as a scatter plot, with each point being a measure of relative gene expression per subject for both studied groups. Horizontal lines represent mean value ± standard deviation. Data were analysed using the Mann–Whitney U test, with *p*-values below 0.05 as significant. ns stands for not significant.

Relative gene expression data of seven miRNAs were compared between suicide completers and control group subjects. While none of the seven tested miRNAs reached statistical significance, two of them showed a trend towards statistical significance (hsa-miR-4516 with *p*-value 0.0998 and hsa-miR-381-3p with *p*-value 0.0718). Gene expression of both miRNAs was on average higher in suicide completers compared to the control group subjects. Detailed statistical information is presented in Table 4. Graphical representation of miRNA expression results can be found in Figure 1.

### 3.2. Target Gene mRNA Expression

**Figure 2:**
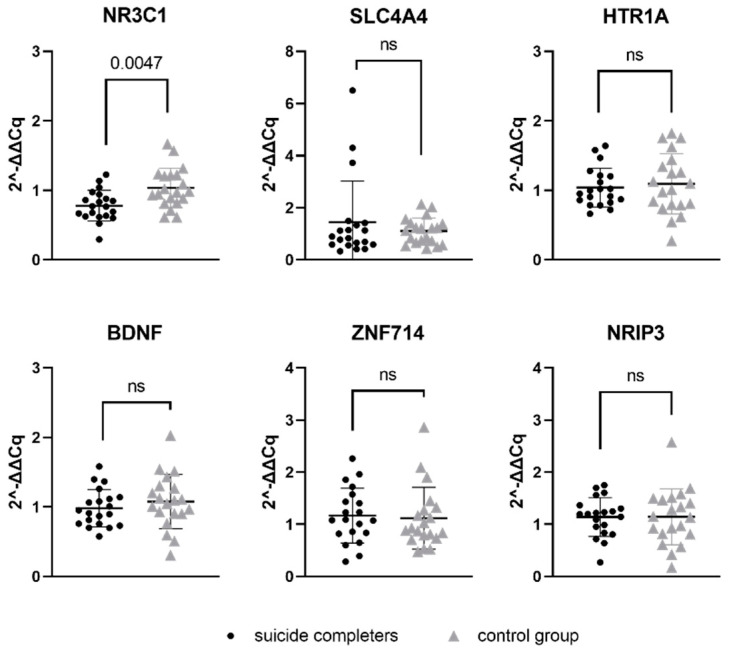
Relative gene expression of target gene mRNAs in Brodmann area 10. Data are presented as a scatter plot, with each point being a measure of relative gene expression per subject for both studied groups. Horizontal lines represent mean value ± standard deviation. Data were analysed using Mann–Whitney U test, with *p*-values below 0.05 as significant. ns stands for not significant.

Relative gene expression data of six genes, which represent a potential target for previously mentioned seven miRNAs, were compared between suicide completers and control group subjects. One of the studied genes, *NR3C1*, reached statistical significance (*p*-value 0.0047). Gene expression of *NR3C1* was lower in suicide completers compared to control group subjects. Detailed statistical information are presented in Table 5. Graphical representation of mRNA expression results can be found in Figure 2.

## 4. Discussion

Disposition to suicidality is determined by genetic as well as by environmental factors, while one of the main connecting links between these two factors could be epigenetics [15]. Numerous studies exist, connecting epigenetic mechanisms with suicidal behaviour, with DNA methylation being predominately studied [15]. With our study, we aimed to design an algorithm that could efficiently select miRNAs that potentially target our genes of interest, and analyse whether or not the selected miRNAs exhibit altered expression levels in the brain of suicide completers.

We selected miRNAs using integrated data of five miRNA databases/web algorithms: DIANA microT-CDS, miRDB, miRmap, miRWalk and TargetScan. We developed a custom sorting algorithm that ranked miRNAs into an order according to the probability of target gene interaction and potential expression in the brain. For each miRNA, a total score was calculated by combining interaction scores and expression scores. Using qPCR, we tested the association between expression of miRNAs (hsa-miR-4516, hsa-miR-3135b, hsa-miR-124-3p, hsa-miR-129-5p, hsa-miR-27b-3p, hsa-miR-381-3p, and hsa-miR-4286) and their potential target mRNAs (*SLC6A4*, *HTR1A*, *BDNF*, *NR3C1*, *ZNF714*, and *NRIP3*) that have been previously recognised as important candidate genes for suicidality. None of the seven studied miRNA reached statistical significance of *p*-value below 0.05. However, two of the studied miRNAs, hsa-miR-4516 and hsa-miR-381-3p, did show a trend towards statistical significance with the *p*-value below 0.1.

Interestingly, according to our algorithm, hsa-miR-4516 was the highest ranked miRNA, with a strong association to *SLC6A4* gene. Experimental data showed a high expression rate in the brain of our subjects, compared to the expression rate of the other six miRNAs we analysed. Hsa-miR-4516 has already been described in the literature as associated with mental disorders. In subjects diagnosed with bipolar disorder, there was an increased level of hsa-miR-4516 expression in peripheral blood exosomes [40]. The second miRNA we observed with a trend towards significance, hsa-miR-381-3p, has not yet been associated with suicidality. A study did, however, observe hsa-miR-381-3p as potentially involved in brain state and inflammation, where a decreased expression of has-miR-381-3p was observed in the serum of patients with Alzheimer’s disease [41]. Taking both hsa-miR-4516 and has-miR-381-3p into account, together they target too few genes to look at a common gene ontology assessment. This is the reason that we looked at the gene ontology of hsa-miR-4516 and hsa-miR-381-3p separately. Both miRNAs have numerous other target genes in addition to the six target genes that we selected in our analysis [42]. Hsa-miR-4516 appears to be targeting genes involved in cell adhesion and synaptic signalling, while hsa-miR-381-3p targets genes involved in transcription regulation and neurogenesis [42]. The altered expression of miRNAs could therefore have a broader effect on the cell functioning. 

The remaining five miRNAs that we analysed (hsa-miR-3135b, hsa-miR-124-3p, hsa-miR-129-5p, hsa-miR-27b-3p, and hsa-miR-4286) showed no difference in expression levels between suicide completers and control group subjects. Of these, hsa-miR-124-3p appears to be the most studied in regard to brain states. It belongs to the miR-124 family, which is highly neuron-specific and it is thought to be important for the functioning and development of the nervous system (however, it so far lacks sensitivity to be a useful biomarker) [43,44]. Hsa-miR-124-3p has been associated with depression-like states. Cell and animal models indicate that miR-124-3p binds to the 3′UTR region of *NR3C1*, leading into decreased levels of *NR3C1* gene expression [45]. Similarly, it is increased in subjects with major depressive disorder (both in living subjects and in those deceased from causes other than suicide) [46]. 

MiRNAs work by binding to target gene mRNA and subsequently decrease the expression of the target gene. Therefore, we hypothesised that if there are present changes in miRNA expression in suicide completers, that the changes in expression could also be observed at the level of the target genes. Our study revealed decreased expression of *NR3C1* gene in suicide completers. *NR3C1* is a gene that codes for glucocorticoid receptor and is, thus, heavily involved in the stress response. The HPA axis and its dysfunction is known to be an important risk factor for suicidal behaviour. Dysfunction of the stress axis and poor adaptation to stress may also be associated with changes in epigenetic regulation and gene expression of involved genes. Cortisol, also known as the stress hormone, binds to the glucocorticoid receptor, which has an anti-inflammatory effect. If the number of glucocorticoid receptors is adequate, the stress response is appropriate. However, if the number of glucocorticoid receptors is reduced, the stress response can be prolonged [47]. In animal studies, the separation of a pup from its mother is associated with increased DNA methylation in the *NR3C1* promoter region. This leads to reduced expression of the *NR3C1* gene and, consequently, a more pronounced response of the organism to stress [48]. Similarly, hypermethylation on *NR3C1* promoter in downregulated gene expression was determined to be more likely in suicidal patients with a major depressive disorder compared to in the control subjects [49]. Numerous mechanisms exist inside the cell which can influence gene expression. Studies show that miRNA can also greatly affect the glucocorticoid response system [50], which could lead us to believe that one possible explanation for a decreased level of *NR3C1* mRNA in suicide completers could be the increased expression of has-miR-4516 ahashsa-miR-381-3p. Decreased levels of *NR3C1* mRNA have already been associated with suicidal behaviour [17,51,52].

The first miRNAs were discovered in the 1990s, and about a decade later, they have been adopted as a separate class of biological regulators. Through numerous studies, miRNAs were recognised as one of the most important mechanisms for gene expression regulation [22,23,53]. This is also the reason why today miRNAs are tested as biological markers to be used in the diagnosis and prognosis of various neuropsychiatric disorders [54]. The great potential of miRNAs for clinical medicine is their presence and biological activity not only in the cell they were generated in, but also in other, neighbouring cells or distant tissues. One of the mechanisms for miRNAs transportation are extracellular vesicles (EVs) [28,55].

Although miRNAs appear to be a promising biomarker, there are some limitations associated with their study. One of the main limitations at the stage of miRNA selection is the non-uniform nomenclature of miRNAs between different data sources. This was an important issue in our study, as we used five different data sources/web algorithms to extract the data on miRNAs. We treated all the miRNAs with the same sequence as identical (for example, hsa-mir-125b-1-3p and hsa-mir-125b-2-3p have the same sequence), as the miRNA sequence was the most important factor in databases/web algorithms.

The limitation of our study is the sample size, which was rather small; on the other hand, the inclusion criteria were rather strict, resulting in a homogenous sample in which even smaller changes could be more likely detected. The significant difference in age between suicide completers and control group subjects also needs to be taken into consideration. The difference in age can be attributed to the cause of death. Middle-aged men tend to be most at risk of suicide, while the risk of sudden cardiac death tends to increase with age. If subjects over the age of 65 would be included, the age difference between the two groups examined would be possibly lower (due to older suicide completers), but this could then present another problem as after the age of 65, age-related neurodegeneration can start occurring. Still, subjects who died from sudden cardiac arrest present a control group with a high homogeneity within the group. In the end, we have to be careful with the interpretation of the results. Namely, miRNAs represent just one of the possible levels of gene expression regulation. Therefore, if a miRNA is determined as the regulatory miRNA based on a database/web algorithm, its role in the particular physiologic state of a cell still has to be demonstrated, suggesting the importance of coordinated use of in silico and experimental procedures.

More general limitations are related to the studies on miRNAs in association with mental disorders and suicide, as there is a lot of overlap in the nature of both phenotypes. Currently, there is a clear lack of studies in which the result of miRNAs expression could be associated only to suicidality; therefore, we can more or less treat the miRNAs as the biomarkers for mental disorders, and not really just as the biomarkers for suicidality specifically.

While the algorithm had many beneficial properties, such as integration of five different databases and gene expression data integration, the results of the expression of the identified miRNAs were not significant when comparing suicide completers and controls. This could be due to various reasons such as the selection of candidate genes and algorithm weaknesses which have to be further identified and improved.

## 5. Conclusions

The aim of our study was to confirm the listed miRNAs as potential biomarkers for suicide, as miRNAs are, in addition to being an important gene expression regulator, also very stable molecules in terms of their chemical stability (higher resistance to RNase degradation, pH and temperature changes), with tissue-specific expression, and could, therefore, also be a suitable biomarker from a technical point of view. Because of their characteristics and their presence in body fluids, miRNA testing in the future could also advance to the clinical setting. Namely, one of the important cargos of the EVs are miRNAs. It has been shown that EVs can cross the blood–brain barrier [56], thus bringing the information from the central nervous system to the periphery (e.g., blood), where sampling can be performed with minimally invasive protocols. Therefore, miRNAs represent a great potential for central nervous system biomarkers for tackling the brain’s (patho)physiological status.

## Figures and Tables

**Table 1:** Subject information and group comparison, including age, postmortem interval and RNA integrity number (mean ± SD).

	Suicide Victims	Control Group	*p*-Value
Age (years + SD)	44.6 ± 10.4	54.5 ± 7.4	0.0016, t = 3.391, df = 38
PMI (hours + SD)	27.5 ± 14.0	27.1 ± 21.6	0.9430, t = 0.07202, df = 38
RIN	7.7 ± 0.22	7.26 ± 0.28	0.0794, t = 2.557, df = 8

**Table 2:** Number of predicted miRNAs involved in each target gene regulation.

Database	*BDNF*	*HTR1A*	*SLC6A4*	*NR3C1*	*ZNF714*	*NRIP3*
miRWalk	2353	1462	1942	2349	1858	1916
miRmap	979	48	1300	370	65	1202
TargetScan	775	153	1115	976	1506	875
DIANA microT-CDS	272	1	201	301	303	211
miRDB	191	21	161	472	269	163

**Table 3:** Final miRNA selection. MiRNAs were selected by an in silico algorithm. The table contains separated values of target gene analysis and expression analysis, as well as combined values.

Selected miRNAs	Target Interaction Analysis	Expression Analysis	Weighted Sum
hsa-miR-4516	13.63	17.17	30.80
hsa-miR-3135b	16.75	13.57	30.33
hsa-miR-124-3p	11.55	17.87	29.43
hsa-miR-129-5p	20.89	8.47	29.35
hsa-miR-27b-3p	15.80	12.34	28.14
hsa-miR-381-3p	20.01	7.48	27.49
hsa-miR-4286	9.37	18.06	27.43

**Table 4:** Relative gene expression of miRNAs between suicide completers and control group subjects, determined using the Mann–Whitney U test.

Selected miRNAs	Suicide Completers Median	Control Group Median	U Test Statistic	*p*-Value
hsa-miR-4516	1.738	1.092	109	0.0998
hsa-miR-3135b	1.002	0.8698	187	0.7381
hsa-miR-124-3p	1.520	0.8713	157	0.2534
hsa-miR-129-5p	1.184	0.9738	170	0.4291
hsa-miR-27b-3p	1.167	1.044	171	0.6071
hsa-miR-381-3p	1.468	0.9845	133	0.0718
hsa-miR-4286	1.266	1.065	152	0.2012

**Table 5:** Relative gene expression of target gene mRNAs between suicide completers and control group subjects, determined using the Mann–Whitney U test.

mRNAs	Suicide Completers Median	Control Group Median	U Test Statistic	*p*-Value
*NR3C1*	0.7685	0.9780	97	0.0047
*SLC6A4*	0.8601	1.163	179	0.5831
*HTR1A*	0.9735	1.054	188	0.7584
*BDNF*	0.9309	1.071	161	0.3013
*ZNF714*	1.085	0.9057	177	0.5468
*NRIP3*	1.194	1.143	192	0.8410

## Data Availability

Available upon request.

## Supplementary Materials

The following supporting information can be downloaded at: https://www.mdpi.com/article/10.3390/genes13040562/s1, Table S1: Study subject information.
