# Supplementary material for: Suicide and Changes in Expression of Neuronal miRNA Predicted by an Algorithm Search through miRNA Databases
Source: Genes (Basel). 2022 Mar 23;13(4):562. doi: 10.3390/genes13040562 (PMC9025835; doi:10.3390/genes13040562)
Supplement: Supplementary file 1 [file genes-13-00562-s001.zip › genes-1627495-supplementary.pdf]

genes

MDPI

Supplementary

Suicide and changes in expression of neuronal miRNA predicted by an algorithm search through miRNA databases

Alja Videtič Paska [1], Urban Alič [2], Tomaž Zupanc [3] and Katarina Kouter [1,*]

[1] Institute of Biochemistry and Molecular Genetics, Faculty of Medicine, University of Ljubljana, 1000 Ljubljana Slovenia; alja.videtic@mf.uni-lj.si, katarina.kouter@mf.uni-lj.si
[2] Faculty of Medicine, University of Ljubljana, 1000 Ljubljana, Slovenia; urbanalich@gmail.com
[3] Institute of Forensic Medicine, Faculty of Medicine, University of Ljubljana, 1000 Ljubljana, Slovenia; tomaz.zupanc@mf.uni-lj.si
* Correspondence: katarina.kouter@mf.uni-lj.si

Table S1. Study subject information Toxicology screen was performed on blood and urine.

Subjects	Cause of death	Age (years)	PMI (hours)	Toxicology screen
S1	hanging	38	12	none detected
S2	hanging	60	22.5	none detected
S3	hanging	39	28	none detected
S4	hanging	46	9,5	antidepressants, antipsychotics
S5	hanging	54	36.5	ethanol
S6	hanging	51	15	ethanol, antidepressants, anxiolytics
S7	hanging	59	15	antidepressants
S8	hanging	58	41,5	ethanol
S9	hanging	50	12	ethanol
S10	hanging	35	51.5	ethanol
S11	hanging	32	61	ethanol
S12	hanging	48	40	antidepressants
S13	hanging	54	23	none detected
S14	hanging	39	33	ethanol
S15	hanging	32	32.5	ethanol
S16	hanging	46	36	ethanol
S17	hanging	58	17	ethanol
S18	hanging	30	13	ethanol
S19	hanging	29	34.5	ethanol, antipsychotics
S20	hanging	33	16.5	none detected
C1	sudden cardiac arrest	51	15	none detected
C2	sudden cardiac arrest	54	19	none detected
C3	sudden cardiac arrest	47	12	none detected
C4	sudden cardiac arrest	55	17	none detected
C5	sudden cardiac arrest	60	48	none detected
C6	sudden cardiac arrest	50	20	ethanol
C7	sudden cardiac arrest	45	37	none detected
C8	sudden cardiac arrest	59	22	none detected
C9	sudden cardiac arrest	57	28	none detected

C10	sudden cardiac arrest	52	17	none detected
C11	sudden cardiac arrest	61	18	none detected
C12	sudden cardiac arrest	50	15	none detected
C13	sudden cardiac arrest	33	16	none detected
C14	sudden cardiac arrest	60	13	ethanol
C15	sudden cardiac arrest	49	59	antiepileptics, antipsychotics
C16	sudden cardiac arrest	60	106.5	ethanol
C17	sudden cardiac arrest	64	17	none detected
C18	sudden cardiac arrest	63	23	ethanol
C19	sudden cardiac arrest	57	19	none detected
C20	sudden cardiac arrest	63	20	ethanol

S – suicide victim, C- control group subject, PMI- postmortem interval
